# Ultrasound-assisted immunotherapy for malignant tumour

**DOI:** 10.3389/fimmu.2025.1547594

**Published:** 2025-05-13

**Authors:** Xiaowen Cai, Yujie Liu, Guosheng Luo, Zhanwang Yu, Cheng Jiang, Chuanshan Xu

**Affiliations:** ^1^ School of Applied Biology, Shenzhen City Polytechnic, Shenzhen, China; ^2^ Guangzhou Municipal and Guangdong Provincial Key Laboratory of Molecular Target & Clinical Pharmacology, the NMPA and State Key Laboratory of Respiratory Disease, School of Pharmaceutical Sciences, Guangzhou Medical University, Guangzhou, China

**Keywords:** ultrasound, immunotherapy, malignant tumour, ablation, ultrasound-targeted contrast agents, sonodynamic therapy

## Abstract

Malignant tumour represents a significant global public health concern. The advent of immunotherapy has brought about a revolutionary shift in the landscape of tumour treatment, offering a ray of hope to patients across the globe. Immunotherapy strategies have demonstrated considerable promise in clinical trials. However, the immunosuppressive environment within the tumour microenvironment has constituted a significant obstacle to the advancement of immunotherapies. It is therefore imperative to develop more efficacious and personalised approaches. The utilisation of non-invasive ultrasound-assisted immunotherapy represents a promising strategy. Ultrasound has the capacity to induce an immune response and stimulate other drugs to achieve a specific response, thereby reducing the toxic side effects of treatment and enhancing the outcome of immunotherapy. This paper presents a systematic introduction to the various mechanisms related to ultrasound and reviews the recent advancements of ultrasound-assisted tumour immunotherapy, including ultrasonic ablation, combined application with contrast agents, and sonodynamic therapy.

## 1 Introduction

In the 21st century, malignant tumour represents a significant threat to human health (1). The high morbidity and mortality associated with malignant tumour have attracted sustained attention and investment in basic and clinical research. Currently, surgical, radiotherapeutic and chemotherapeutic interventions are the three principal modalities for the treatment of neoplastic disease, with the capacity to exert a significant inhibitory effect on tumour growth (2–5). However, these conventional treatments have limited outcomes, especially after tumour metastasis. It is therefore of particular importance to seek improved methods of treating tumours.

The rapid development of molecular biology has led to the discovery that the tumour microenvironment plays a pivotal role in the initiation and progression of tumours (6). The treatment of tumours is increasingly taking the entire microenvironment into account, with immunotherapy representing a significant treatment method for the immune system of the microenvironment (7). Tumour cells within the microenvironment demonstrate deficiencies in antigen presentation mechanisms, enhanced negative regulatory pathways and the recruitment of immunosuppressive cells to evade immune surveillance (8, 9). This results in the inhibition of effector functions of immune cells and the termination of anti-tumour immune responses. It is for this reason that tumour immunotherapy has emerged as a means of activating or enhancing the body’s own immune response in order to achieve tumour killing without affecting normal cells (10). Moreover, in cases of intractable tumour metastasis, immunotherapy has been shown to have a positive therapeutic effect on distal tumours, thus offering a novel approach to the treatment of tumour (11). However, due to the intricate nature of the tumour microenvironment, variations in immune cell infiltration and tumour heterogeneity, it has been observed that patients exhibit disparate responses to immunotherapy, which introduces ambiguity regarding the efficacy of immunotherapy (12).

**Figure 1:**
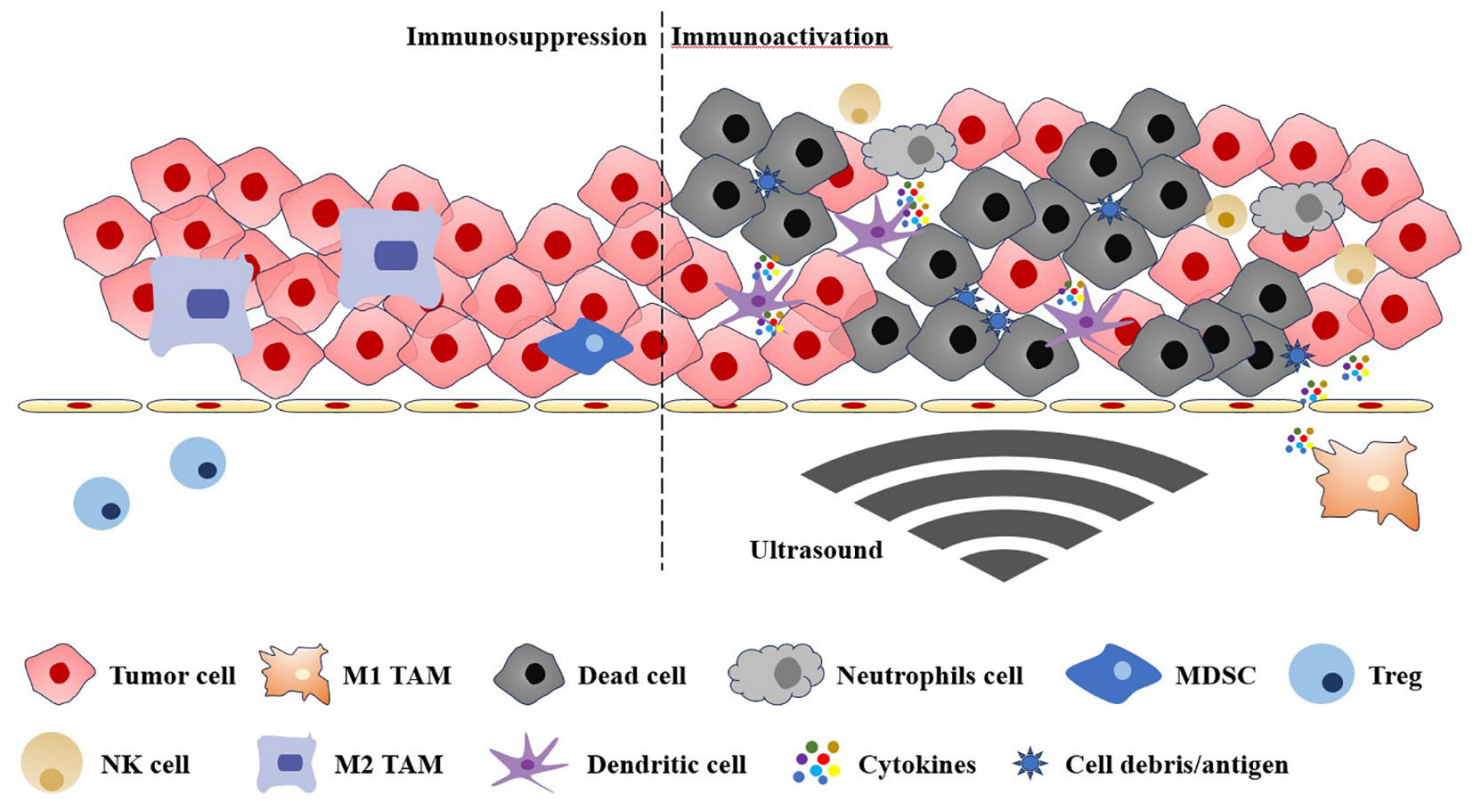
The immunosuppressive and immunoactive tumour microenvironments are regulated by the ultrasound. TAM, tumour-associated macrophages; MDSC, myeloid-derived suppressor cell; Treg, regulatory T cell; NK cell, natural killer cell.

In order to enhance the therapeutic efficacy and mitigate the adverse effects, researchers have concentrated their efforts on the integration of immunotherapy with alternative therapeutic modalities (13). The combination of physical therapy, chemotherapy, gene therapy and immunotherapy has demonstrated synergistic effects and complementary advantages, thereby improving treatment outcomes (14, 15). In particular, physiotherapy and chemotherapy have been demonstrated to possess the capacity to diminish tumour volume, stimulate tumour immunogenicity, facilitate the release of inflammatory cytokines, antigens, and recruit immune effector cells (16, 17). Recently, ultrasound therapy as one of the common physiotherapies are paid attention because ultrasound treatment has been observed to possess potential immune-activating properties in the context of these ultrasound studies, thereby conferring additional benefits to ultrasound-assisted tumour immunotherapy (18–24). The application of ultrasound has been demonstrated to enhance the immunogenicity of tumours through a combination of thermal, cavitation and mechanical effects within biological tissues. This process facilitates the permeation of antigens through the infiltration barrier, effectively converting “cold” (low infiltration) tumours into “hot” (high infiltration) tumours, thereby potentiating the efficacy of immunotherapy (25). Furthermore, the great penetration of ultrasound wave can be exploited to achieve precise delivery of immune agents to sufficient depths, thereby enhancing drug accumulation at targeted sites and ensuring efficient immunotherapy (26). A comprehensive description of ultrasound-induced antitumour immunity can be found in **Figure 1**. Tumour immunotherapy is a complex process involving antigen presentation, the migration of immune cells, and the activation of effector cells (27, 28). As an externally controllable stimulus source, ultrasound can act as a multiple immune linker in tissues through thermal and mechanical effects to render patients sensitive to immunotherapy. Firstly, ultrasound can facilitate the process of immunotherapy within the body. For example, sonication can destroy the compression blood vessels of tumour, relieve the activity restriction of immune cells, and increase the infiltration of immune cells in the tumour site (29). Secondly, ultrasound treatment can also induce immunogenic cell death (ICD) or necrosis in tumours through two distinct mechanisms: mechanical ablation or the obstruction of tumour blood supply. Consequently, the release of tumour-associated antigens and damage-associated molecular patterns is facilitated, thereby enhancing the antigen presentation process. Furthermore, the study demonstrated that ultrasonic cavitation can induce dendritic cells (DCs) maturation, thereby enhancing the antigen presentation process. In summary, ultrasound shows great potential in boosting immunotherapy (30). Accordingly, this review will provide a comprehensive overview of the mechanisms of ultrasound-assisted tumour immunotherapy and the recent advancements in this field.

## 2 Mechanism of ultrasound-assisted immunotherapy for malignant tumour

Frequencies above 20 kHz, designated as ultrasound, exceed the range of human hearing and are extensively employed in clinical settings (31). The most prevalent application of ultrasound is in ultrasound diagnosis. Some medical diagnoses can be completed with or without the use of a contrast agent, and it can also be employed as an auxiliary means of treatment, such as in the case of bone and joint and tumour treatment (32–37). In recent years, there has been a notable advancement in the field of anti-tumour immunotherapy. Ultrasound, as a controllable mechanical wave, has also made significant advancements in the field of ultrasound-assisted tumour immunotherapy (38). It is well-known that the efficacy of cancer immunotherapy is constrained by factors such as immunosuppressive cell invasion and/or upregulation of immune checkpoint expression, cancer cell heterogeneity, and lack of antigen presentation (39). Consequently, administration of immune agents against tumours with monotherapy may yield poor patient benefits (40). However, ultrasound has been shown to effectively regulate the microenvironment of tumour immunosuppression, transform “cold” tumours into “hot” tumours, and enhance the efficacy of immunotherapy therapy (41). The role of ultrasound in this process can be mainly classified into two categories: thermal effect and mechanical (non-thermal) effect. As illustrated in **Table 1**, the thermal effect and mechanical (non-thermal) effect are all outlined therein.

**Table 1:** Differences between different mechanisms of ultrasound.

Mechanism	Primary Driver	Outcome	Key Immunological Effect	Example Application
Thermal effect	Heat	Tumour tissue necrosis or tumour cell apoptosis	ICD, antigen release	Ultrasonic thermal ablation
Mechanical (non-thermal) effects	Mechanically destroy tissue without causing coagulative thermal damage	Mechanically disrupt cells into a homogenate of subcellular debris	Generate more natural conformations compared to those exposed to heat, which are efficient antigens for DCs activation and subsequent cytotoxic T lymphocyte	Histotripsy, Boiling histotripsy
	Cavitation effect	Strong mechanical stress, shockwaves and microjets	ICD, release of inflammatory factors	Sonodynamic therapy

### 2.1 Thermal effect

The thermal effect is the result of the absorption of ultrasonic sound energy into heat by living organisms (42). High-intensity ultrasound (e.g., >10 W/cm^2^) can induce thermal and mechanical stresses in biological tissues (43). Through the modulation of excitation amplitude, pulse duration, and frequency, the thermal stress can elevate tissue temperature more than 55°C. It is widely accepted that temperatures in excess of 55°C induce coagulative necrosis, resulting in immediate cell death (44). Furthermore, maintaining the thermal effect of ultrasound at a temperature that does not cause tumour necrosis, such as 43°C, can increase infiltration of immune cells and immune activation. and mild hyperthermia in this region can induce a reduction in interstitial fluid pressure and an increase in cell membrane fluidity and the production of heat shock proteins (HSP) (45–48). These proteins have been shown to mediate a range of immune responses in peripheral tumour models (49). By exposing antigenic peptides to macrophages and DCs, HSP triggers an inflammatory response, resulting in the release of inflammatory cytokines and co-stimulatory molecules, thereby inducing a tumour immune response (50).

### 2.2 Mechanical (non-thermal) effects

In addition to thermal effects, any effect that produces biological effects without causing significant warming (1°C above physiological temperature) is collectively referred to as a mechanical (non-thermal) effect. This non-thermal effect is generally believed to be associated with the cavitation effect (31). It is evident that in addition to the cavitation effect, non-cavitation effects such as radiation pressure, radiation torque and sound flow are also associated with mechanical effects. Consequently, the mechanical (non-thermal) effects engendered in disparate application scenarios (e.g. ultrasonic parameter settings) vary. The ensuing mechanisms of action are delineated as follows: mechanical ablation and contrast media-assisted cavitation.

#### 2.2.1 Mechanical ablation

In comparison with thermal ablation, mechanical ablation has been demonstrated to offer enhanced precision in the execution of regional ablation. Moreover, studies have indicated that this method can further stimulate the immune response, a phenomenon that may be attributed to the absence of degenerative antigen proteins *in situ* at the sonication lesion. Two methods of non-thermomechanical tissue ablation using high pressure, pulsed focused ultrasound are histotripsy and boiling sectioning (51).

Histotripsy represents a non-invasive ultrasonic ablation technique that employs cavitation to mechanically disintegrate tissue into acellular fragments. The technique utilizes high-pressure (>15 MPa) ultrasound pulses with extremely low duty cycles (<5%), delivering ultra-short (microsecond to millisecond duration) acoustic bursts to induce controlled cavitation at the focal zone. These precisely generated microbubbles undergo rapid cyclic expansion and violent collapse, producing mechanical stresses that liquefy target tissue into demarcated lesions through extracellular matrix disruption (52). The application of varying parameters, including pulse intensity, duty cycle, and pulse number, can result in the generation of disparate mechanical effects within the tissue (53). The mechanical effects of ultrasound can be broadly categorised as follows: 1) The generation of steam bubbles in the target tissue, which then vibrate and rupture, resulting in mechanical separation and damage to the tissue (54); 2) The tissue is subjected to mechanical destruction, resulting in cell homogenate, following the generation of steam bubbles (55). In comparison to fibrous scar tissue formed as a result of thermal effects, the generation of tissue emulsification through histotripsy is a more conducive approach to immune system activation (56, 57).

Boiling histotripsy (BH) is an alternative histotripsy regime. In comparison to histotripsy, BH utilises lower peak pressures and longer pulses, thereby significantly addressing the size, focusing gain and frequency requirements of the high-intensity focused ultrasound (HIFU) source in histotripsy’s application (58). In highly aggressive melanoma models that exhibit a paucity of T lymphocyte (T cell) infiltration, the implementation of a sparse scan protocol (1 mm spacing between sonications) has been shown to induce B cells, macrophages, monocytes, granulocytes, and both conventional DCs subsets (i.e. cDC1s and cDC2s) (59). These cells have been observed to acquire antigen in a markedly enhanced manner. The proportion of antigen-positive immune cells in tumour lymph nodes increased almost threefold 24 hours after treatment. Furthermore, BH treatment has been demonstrated to induce ICD, thereby instigating anti-tumour immunity. Consequently, the employment of BH is anticipated to address the challenge posed by the low immunogenicity of tumours in immunotherapy.

#### 2.2.2 Contrast agent-assisted cavitation

The term ‘cavitation effect’ is used to describe the process by which minute bubbles (referred to as a ‘cavitation core’) within a liquid are subject to vibration, growth and the gathering of acoustic field energy under the influence of sound waves. Once the energy reaches a specific threshold, the cavitation bubbles undergo a sudden collapse and closure (60). Given that bubbles with a radius of less than 1 mm are readily dissolved, the cavitation effect is typically assisted by contrast agent such as microbubbles. The use of contrast agent has been demonstrated to result in the damage of endothelial cells through the generation of microflows, microjets and free radicals by ultrasonic cavitation. The use of contrast agent-assisted cavitation at the tumour site has been demonstrated to cause microvascular rupture and tumour cell apoptosis. Furthermore, this process has been shown to hinder tumour angiogenesis, enhance the effect of immunotherapy and regulate the tumour immunosuppressive microenvironment (61, 62). In addition to the immune activation effect brought about by ultrasound itself, ultrasound can also be used as a stimulus for drug delivery to synergistically activate immunity (63–66). In tumour tissues, vasculature is not fully developed so that enhanced the permeability and retention effect (EPR effect) exists, which facilitates drug delivery to the tumour site. However, it has been found in clinical trials that patients do not benefit much from EPR effect because of the high pressure inside the tumour tissues. Passive drug delivery often cannot achieve effective delivery to the tumour site. Ultrasound can improve the delivery efficiency of drugs mainly through thermal and non-thermal effects. Some studies have shown that the assistance of ultrasound can disrupt the order of membrane molecules to improve the delivery efficiency of macromolecules such as immunomodulators (67). In addition, ultrasound can open the blood-brain barrier or vascular tumour barrier and increase the expression of immune-related molecules such as proinflammatory cytokines at the tumour site in addition to facilitating the delivery of macromolecular drugs to the brain tumour site (68, 69).

## 3 Application of ultrasound-assisted immunotherapy for malignant tumour

The immune system plays a significant role in the pathogenesis and progression of tumours (70). The correct identification and destruction of tumour cells that have undergone mutation from normal cells by the immune system is referred to as immune surveillance (71). This process necessitates the involvement of antigen-presenting cells, which are responsible for capturing tumour-associated antigens, secreting cytokines and chemokines, activating T cells, and ultimately eradicating tumour cells through T cells. Concurrently, the toxic reaction generated by T cells will also result in the production of novel antigens, thereby initiating the subsequent phase of the immune cycle response (72). However, tumour tissue can evade the immune system through a variety of mechanisms, including the elimination of functional DCs and the inhibition of their key functions, thereby creating an immunosuppressive tumour microenvironment (8). In the late 1890s, William B. Coley proposed tumour immunotherapy based on the prevailing immunosuppressive conditions within the tumour microenvironment (73). This approach utilises the body’s immune system to specifically identify and destroy tumours. The benefits of this therapeutic approach are that it causes minimal damage to surrounding tissues and can induce immune memory, thereby enabling the body to maintain its anti-tumour defences (74). The current methods for achieving tumour immunity can be broadly classified into three categories: immune checkpoint inhibitor, tumour vaccines and adoptive cell transfer (75). As an adjuvant therapy, it has been demonstrated that patients with advanced tumours derive benefit from these therapies (76). Nevertheless, the effectiveness of immunotherapy is constrained by the existence of numerous immune evasion pathways. Consequently, there is a pressing need for the development of a reliable method to enhance immune function, in order to address the shortcomings of current immunotherapy. As a non-invasive physical technology, ultrasound has reinvigorated tumour immunotherapy with its safety and efficacy. Ultrasound has the potential to regulate tumour immune processes in a multitude of ways (77). For instance, tumour fragments released *in situ* following HIFU treatment can be conceptualised as antigens for the immune system, which can be employed as an *in-situ* vaccine to stimulate a systemic immune response. A number of clinical studies have also demonstrated that patients after HIFU treatment exhibit enhanced immune responses (78–81). Nevertheless, the capacity of ultrasound-activated immunity to diminish tumour cells and diminish substantial tumour masses is constrained, and the commencement of immunity necessitates an interval of time. Consequently, the combination of alternative methods for rapid tumour destruction and the utilisation of the memory effect of immunity for tumour treatment not only reduces the required therapeutic dose but also minimises the potential for adverse effects, which is of clinical significance. At the present time, ultrasound-assisted tumour immunotherapy is a rapidly developing field of research. The next will introduce the latest developments in the use of ultrasonic ablation to enhance tumour immunotherapy, ultrasound combined with contrast agents for tumour immunotherapy, and the use of sonodynamic therapy (SDT) to achieve tumour immunotherapy.

### 3.1 Ultrasonic ablation

Ultrasonic ablation is a method of utilising the thermal or mechanical effects of ultrasound to induce apoptosis and necrosis of tumour cells, thereby reducing tumour volume and achieving tumour treatment. It represents a significant avenue for patients to pursue localised treatment (82). It has been demonstrated that ultrasonic ablation can induce the upregulation of potent innate immunity tools, namely HSP, which increase tumour immunogenicity (83). Furthermore, it has been demonstrated that cell debris and tumour-associated antigens released by ultrasonic ablation can be recognised by the immune system, thereby activating immunity (84). However, the majority of current ultrasonic ablation applications were based on the thermal effects. Additionally, recent studies have employed short bursts (lasting from microseconds to milliseconds) at high pressures (>15 MPa) and with a low duty cycle (<5%), a method known as histotripsy (85). This technique utilises high-pressure ultrasonic pulses to mechanically separate and emulsify tissue into a liquid, decellularised, homogeneous substance. The liquefied tissue homogenate is fully absorbed by the body, resulting in minimal residual fibrous tissue. Furthermore, the mechanical ablation demonstrated greater DCs activation than thermal ablation, suggesting that the histotripsy technique may represent a promising approach for tumour ablation in conjunction with immunotherapy (86, 87). The destructive capacity of ultrasound in the context of tumour destruction is contingent upon the presence of high acoustic pressure. However, it should be noted that ultrasound attenuates during the sound beam penetration of thick tissue, and the acoustic window of ultrasound is affected by gas-bearing organs or bone in the tissue. In order to address the issue of attenuation when ultrasound penetrates substantial tissue, Tang’s group proposed the utilisation of ultrasound needles as a solution (88). They utilised modified ultrasound to target tumours for thermal ablation and mechanical destruction, and combined this technique with anti-PD-L1 antibody immunotherapy in a mouse tumour model. The application of minimally invasive ultrasound needles not only inhibited tumour growth through mechanical ablation, but also increased the infiltration of CD8^+^ T cells in the tumour, alleviated the immunosuppressed tumour microenvironment, induced the systemic anti-tumour immune response, and enhanced the therapeutic effect of anti-PD-1. The study posited that the minimally invasive use of ultrasound may offer novel approaches for the treatment of deep tumours.

It is noteworthy that tumours treated with mechanical ablation, as opposed to thermal ablation, can be conceptualised as an immediate antigen generating pool at the tumour site for utilisation by the immune system (89, 90). The combination therapy using mechanical ablation and immune checkpoint suppression has seen significant development. Abe’s team found that mechanical ablation followed by multiple anti-PD-L1 treatments could effectively activate the systemic anti-tumour immune response and inhibited the growth of distal tumours (91). Pepple’s study also detected strong immune-enhancing responses in mouse melanoma and hepatocellular carcinoma models after administration of anti-CTLA-4, followed by mechanical ablation the next day, and two subsequent anti-CTLA-4 treatments (92). Furthermore, the temporal parameters of antibody immunotherapy in combination with recombination therapy exhibit variation, a factor that may be associated with the divergent tumour immunogenicity and immune microenvironmental characteristics observed (93, 94).

### 3.2 Combined with ultrasound-targeted contrast agents

Microbubbles represent the oldest developed ultrasound-responsive delivery materials as well as extensively as ultrasound contrast agents (95). In the bloodstream, the microbubbles undergo a series of oscillations, including steady expansion and contraction, growth, and finally violent collapse, which is also known as inertial cavitation (96). It is currently believed that the main mechanisms of ultrasound-targeted microbubble destruction-mediated tumour immunity are stable cavitation and Inertial cavitation (97, 98).

#### 3.2.1 Stable cavitation (non-inertial cavitation)

Stable cavitation (also known as non-inertial cavitation) is characterised by the repeated contraction and expansion of microbubbles within a stable fluid environment (99). This process induces a surrounding fluid flow. This microflow exerts shear stress on the cell, resulting in transient permeability of the cell membrane (i.e. acoustic perforation), which is conducive to the delivery of loaded drugs such as immunoactive substances.

In addition to facilitating the delivery of immunotherapy molecules, ultrasonically targeted microbubbles can also induce cell rupture when interacting with ultrasound, which can subsequently elicit a series of biological effects. Dong and colleagues devised a two-part therapeutic regimen (100). One component was a phospholipid microvesicle (C@MBs) loaded with CXC chemokine ligand 10 (CXCL10). The material was capable of opening the blood-brain barrier and releasing CXCL10, which in turn recruits CD^8+^ T cells and promotes their migration and adhesion to tumour tissues when stimulated by 0.4 W/cm² at a low frequency of ultrasound. The second component was the fusion of a mature DCs membrane with a platelet-derived growth factor (PDGF) and a phospholipid-based material containing interleukin-2 (IL-2) and anti-programmed cell death 1 ligand 1 (aPD-L1), designated IP@DCNBs. The release of IL-2 in response to 1.58 W/cm² ultrasound irradiation was observed to reduce the depletion of CD^8⁺^ T cells, while aPD-L1 was seen to enhance the activity of CD^8⁺^ T cells. This strategy employs low-frequency and high-frequency ultrasound to facilitate the sequential delivery of glioblastoma and achieve the “open-source throttling” effect on CD^8⁺^ T cells.

#### 3.2.2 Inertial cavitation

Inertial cavitation exerts a greater pressure on microbubbles, resulting in their collapse and the generation of stronger mechanical forces. This leads to irreversible damage to cells and tissue destruction. Tumour cell injury can result in the production of cell debris and the release of antigens, while vascular tissue injury can enhance antigen diffusion, thereby initiating a subsequent immune response. This includes the induction of DCs to present antigens and the maturation of T cells to upregulate immune functions. It can be concluded, therefore, that inertial cavitation plays a pivotal role in immune activation induced by ultrasound-targeted microbubble destruction.

The team led by Wu employed perfluoropropane to generate low-intensity focused ultrasound-responsive microbubbles (101). In ultrasound conditions of 1 MHz, 3 W/cm², 50% duty cycle, microbubbles were observed to inhibit tumour growth in mice implanted with the 4T1 tumour cell line. This was achieved by blocking blood perfusion and causing tumour cell damage. It has also been demonstrated that ICD induced by cell injury leads to immune activation. An analysis of immune cell populations and cytokines at the tumour site has revealed a significant increase in the proportion of mature DCs and cytotoxic T lymphocytes, accompanied by elevated levels of both IL-12 and TNF-α in serum. Furthermore, microbubbles have been shown to exert a synergistic effect on tumour immunotherapy when combined with anti-PD-L1 in tumour-bearing mice.

In a similar manner, nanobubbles with reduced dimensions have the capacity to augment anti-tumour immunotherapy through the utilisation of ultrasonic cavitation effects. The utilisation of perfluoropentane as the gas core of nanovesicles in the preparation of nanoparticles has been demonstrated to induce a novel form of programmed necrosis, termed caspase-independent programmed necrosis, in mouse tumour models through ultrasonic stimulation (102). Furthermore, the combination of perfluoropentane with immune checkpoint blocking has been shown to result in complete regression of primary tumours. This formulation has been shown to have a favourable therapeutic effect on metastatic tumours in a RIPK3-deficient CT-26 tumour-bearing mouse model, thereby confirming the development potential of ultrasonic-assisted immunotherapy in this field. In addition to nanobubbles, nano-vesicles and nano-sized droplets have also made outstanding contributions in this field. In Hu’ s study, ultrasound-responsive nanovesicles, prepared with perfluoropropane, have been shown to induce tumour cell necrosis through ultrasound-mediated cavitation (103). In a mouse model, the combination of these nanovesicles with anti-PD1 therapy resulted in enhanced systemic anti-tumour immunity and immune memory, as well as a prolonged inhibition of tumour growth and recurrence when compared to the control group. In a study using nanodroplets, lipid shells wrapped the structure of liquid nuclei to achieve the gasification of droplets in specific parts of tumours to provide cavitation nuclei, which were further broken under ultrasonic stimulation to achieve tumour ablation and activate the immune system (104). The immune-activating effects of this combination were also demonstrated in a model of metastatic breast cancer treated with anti-PD1. Ultrasound-targeted contrast agents with nanometre size have been shown to achieve a cavitation effect similar to that of traditional microbubbles (105). In comparison with micron-sized carriers, nano-sized carriers were found to enter tumour tissue through defective blood vessels via the EPR effect, thereby inducing cavitation within the tumour and enhancing the efficacy of immunotherapeutic interventions, particularly in cases of deep-seated tumours (106, 107). Consequently, the utilisation of nanosized ultrasound-targeted contrast agents is anticipated to emerge as a pivotal research trajectory in the forthcoming years. However, the application of ultrasound parameters of different contrast agents is quite different and there is no unified standard, which is also an important reason that limits the application of ultrasound contrast agents in tumour immunotherapy. The application parameters of new contrast agents are shown in **Table 2**.

**Table 2:** Application of ultrasound-targeted contrast agents with tumour immunotherapy.

Contrast agents	Parameters	Ref
Microbubbles	300 W, 10 min on-off cycle 1) 0.4 W/cm^2^ for delivery 2) 1.58 W/cm^2^ for drug release	(100)
Microbubbles	1.0 MHz, 3 W/cm^2^, 50% duty cycle	(101)
Nanobubbles	1 Hz, 30 W, 20% duty cycle	(102)
Nanobubbles	1.0 MHz, 1 W/cm^2^	(103)
Nanodroplet	1) activated at a centre frequency of 3.5 MHz and an MI of 1.84 (PNP of 3.4 MPa) 2) Vesicle bursting at low frequency application at a centre frequency of 105 kHz and an MI of 0.9 (PNP of 290 kPa)	(104)
Nanodroplet	0.65 MHz, 2W/cm^2^, 50% duty cycle	(105)
Nanobubbles	1.0 MHz, 1.5 W/cm^2^, 50% duty cycle	(107)

### 3.3 Sonodynamic therapy

**Figure 2:**
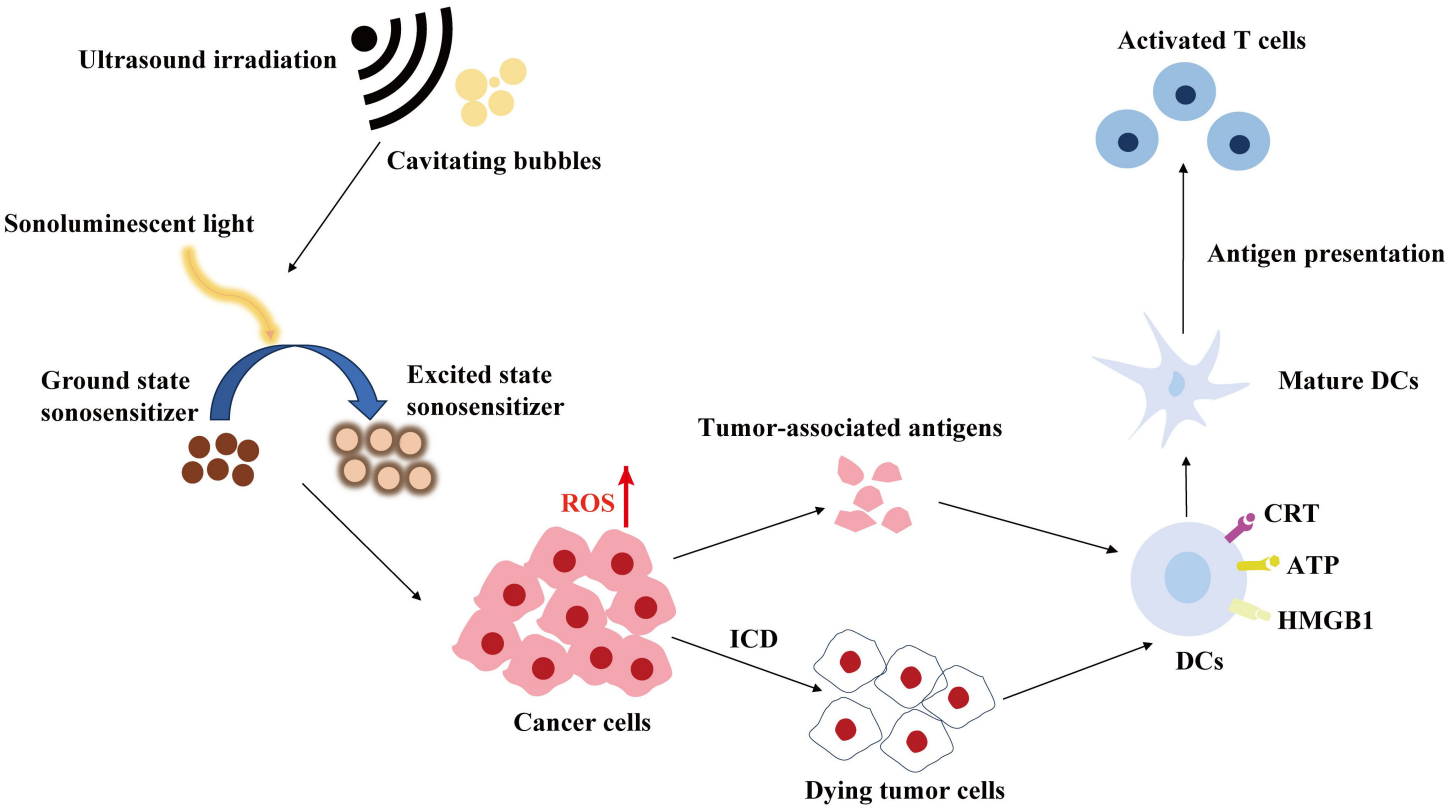
Schematic illustration of SDT and its induced immune effects.

SDT employs ultrasound sensitizers (sonosensitizers) to stimulate the production of active substances, primarily reactive oxygen species (ROS), through ultrasound, thereby facilitating the treatment of tumours (108). A concise illustration of SDT is presented in **Figure 2**. The specific mechanism of ROS production is as follows: the inertial cavitation of sonosensitising agents, induced by ultrasound, causes the collapse of cavitation microbubbles, releasing a significant amount of energy and mediating various sonochemical reactions to generate ROS, including hydroxyl radicals, singlet oxygen and superoxide anion. It has been demonstrated that ROS play a pivotal role in a multitude of immune-related processes (109). Firstly, ROS can induce ICD and the release of damage-associated molecular patterns (DAMPs), including high mobility group box 1 protein (HMGB1), adenosine triphosphate (ATP) and calreticulin (CRT). The maturation of DCs and subsequent immune initiation will eventually result in the transformation of T cells into toxic T cells, thereby achieving tumour killing and long-term immune memory. Furthermore, ROS can reverse the tumour immunosuppressive microenvironment by participating in the maturation of antigen-presenting cells (APCs) and the transformation of anti-inflammatory M2 macrophages into pro-inflammatory M1 macrophages (110, 111). A summary of some of the recent SDT-based tumour immunotherapy options is provided in **Table 3**.

**Table 3:** Application of SDT-based tumour immunotherapy.

Category	Sonosensitizer	Parameters	Ref
Inorganic	Gold nanoparticles	1 MHz	(112)
Organic	Hematoporphyrin	650 kHz, 0.5 W/cm^2^	(113)
Inorganic	Oxygendeficient MoOx (ODM)	1 MHz, 1.5 W/cm^2^, 50% duty cycle	(114)
Organic	Exosome-inhibiting polymeric sonosensitizer (EIPS)	1 MHz, 1.2 W/cm^2^, 50% duty cycle,	(115)
Organic	Chlorin e6 (Ce6)	1 MHz, 2 W/cm^2^, 20% duty cycle	(116)
Inorganic	Schottky heterojunctions containing Sb component (Sb_2_Se_3_@Pt)	30 kHz, 3.0 W/cm^2^	(117)
MOF	Iron-based covalent organic framed nanoadjuvant doped with curcumin and platinum (CFCP)	1 MHZ, 1 W/cm^2^, 50% duty cycle	(118)
Inorganic	Titanium diselenide (TiSe_2_) nanosheets	1 MHz, 0.5 W/cm^2^, 50% duty rate,	(119)
Organic	Protoporphyrin IX (PpIX)	1 MHz, 1.4 W/cm^2^	(120)
Inorganic	Tin monosulfide nanoparticles (SnSNPs)	1 MHz, 1 W/cm^2^, 50% duty cycle	(121)
Organic	Indocyanine green derivatives (IDs)	3.0 MHz, 1.5 W/cm^2^, 50% duty cycle	(122)
Inorganic	Fe-doped TiO_2_ nanodots (Fe-TiO_2_ NDs)	40 kHz, 3 W/cm^2^, 33% duty cycle	(123)
Inorganic	Defect-rich MOF(Ti) (D-MOF(Ti))	1.0 MHz, 50% duty cycle	(124)
Organic	THPP	40 kHz, 2 W	(131)
Organic	Chlorine e6 (Ce6)	1 MHz, 1.5 W/cm^2^, 50% duty cycle	(125)
Inorganic	Fe_3_O_4_@TiO_2_	1 MHz, 1.5 W/cm^2^, 50% duty cycle	(126)
Inorganic	Fluorinated titanium oxide (TiO_2−x_F_x_)	30 kHz, 3 W/cm^2^, 50% duty cycle,	(127)
MOF	Mn-porphyrin-based MOF	40 kHz, 1 W/cm^2^	(128)
MOF	TPP-conjugated porphyrin-based nMOFs (Zr-TCPP(TPP))	1 MHz, 1 W/cm^2^, 50% duty cycle	(129)

Sonosensitizer is an important factor affecting the efficacy of SDT-assisted immunotherapy. The commonly used sonosensitizers in the current SDT mainly included are organic sonosensitizer, inorganic sonosensitizer and organics/inorganics hybrid sonosensitizers.

#### 3.3.1 Organic sonosensitizer

Sonosensitizers of organic molecules can be classified into two main groups: porphyrin derivatives and phthalocyanine. Porphyrin derivatives are the most widely used of them.

The unbalanced REDOX microenvironment at the tumour site, coupled with hypoxia and high reducibility conditions, often impairs the efficiency of SDT in producing ROS. In recent years, there has been a notable increase in interest in multi-functional composite materials (130). In 2022, Yang et al. introduced fluorinated covalent conjugated polymers (COPs) with -5,10,15,20-tetrad (4-hydroxyphenyl) porphyrin (THPP) and perfluorodecanoic acid (PFSEA) as crosslinking agents in acoustic sensitizers (131). The synthesis of THPPF-COP resulted in a material with high sonodynamic efficiency and load capacity for the perfluorinated 15-crown-5-ether (PFCE) model molecule. The application of ultrasound irradiation has been demonstrated to improve blood and lymphatic circulation at the tumour site, thereby alleviating hypoxia. Furthermore, the perfluorocarbon framework provides conditions conducive to high oxygen load, which in turn creates an optimal environment for SDT. The release of injury-related molecular patterns and the occurrence of ICD were confirmed by the detection of calreticulin on the cell surface. This result was corroborated in CT26 tumour-bearing mice, and the subsequent THPPF-COP combined with CD47-mediated immune checkpoint blockade therapy demonstrated robust innate and adaptive anti-tumour immune responses, effectively reversing tumour immunosuppression.

Apoptosis is the primary mechanism of SDT inducing cell death, with the production of ROS playing a crucial role (132). Recently, research has revealed that ROS can also trigger a distinct form of programmed cell death, termed pyroptosis (133). Pyroptosis is a form of programmed cell death driven by inflammatory bodies. It can release more DAMPs and pro-inflammatory factors, which can trigger a stronger immune response than apoptosis. Therefore, inducing pyroptosis may be a more advantageous approach in tumour immunotherapy. Wang and colleagues devised a pyrogenic amplifier comprising the organic acoustic sensitizer chlorine e6 (Ce6), which was coupled to copper tannic acid (CuTA) nanoneedles via an amide bond (125). They found that ultrasonic stimulation could induce Ce6 to release a substantial quantity of singlet oxygen, and CuTA nanoneedles exhibited quadruple enzyme-like activity in response to the tumour microenvironment, thereby exacerbating the REDOX imbalance within the microenvironment. The experimental results demonstrated that the ultrasound-enhanced ROS storm elevated the polarization ratio of M1-type macrophages and effectively induced the maturation of DCs and the subsequent differentiation of killer T cells, thereby enhancing the overall efficacy of immunotherapy.

#### 3.3.2 Inorganic sonosensitizer

Some nanomaterials, comprising inorganic compounds such as noble metal nanoparticles, transition metal oxides and carbon-based nanomaterials, have the capacity to generate reactive oxygen species under ultrasonic stimulation, functioning as sonosensitizers (117). The chemical properties of these inorganic compounds are stable and resistant to photobleaching. In addition, inorganic compounds can be employed as nuclear points to facilitate the generation of ultrasonic microbubbles and augment the cavitation effect.

Hong’s team designed coreshell Fe_3_O_4_@TiO_2_ nanoparticles loaded with VISTA monoclonal antibody (FTV) to target pancreatic ductal adenocarcinoma (PDAC), a checkpoint that plays a role in immune escape from VISTA (126). Given that titanium nanomaterials with a large cavity structure and high specific surface area possess a greater number of catalytic sites, FTV exhibits superior SDT performance compared to commercial TiO₂ (P25) under ultrasonic stimulation. The immunogenic death of cells induced by FTV in both *in vitro* and *in vivo* therapeutic experiments resulted in the release of a pattern of injury-associated molecules that subsequently triggered an anti-tumour immune response in T lymphocytes. Furthermore, the high penetration of ultrasound is an inherent advantage in the treatment of PDAC, given its deep location. The *in vivo* treatment experiment demonstrated that the fibrostitium of the ultrasound combined with the FTV group was significantly loosened, accompanied by an increase in blood vessel density. This resulted in an enhanced delivery of FTV in PDCA, increased accumulation of drugs, and a notable improvement in the efficacy of tumour immunotherapy.

Similarly, the utilisation of inorganic acoustic sensitizers can also facilitate pyroptosis of cells, thereby promoting tumour immunotherapy. Sun and his colleagues devised a novel fluorine-containing titanium oxide acoustic sensitising agent, designated as TiO_2-x_F_x_ (127). The introduction of F atoms into TiO₂ reduces the adsorption properties of the latter to oxygen and water, thereby facilitating the occurrence of acoustic catalytic reactions. Concurrently, the substitution of fluorine for oxygen increases the oxygen vacancy of the acoustic sensitising agent, reduces the band gap and enhances the SDT performance of titanium oxide. *In vitro* and *in vivo* anti-tumour experiments have demonstrated that TiO_2-x_F_x_ is capable of releasing a substantial quantity of ROS in response to ultrasonic stimulation. ROS, in excess, can directly destroy tumour cells and also activate caspase family proteins to induce pyroptosis, reverse immunosuppression and initiate an immune response. Furthermore, pyroptosis has the capacity to engender a robust immune memory effect, thereby preventing tumour recurrence.

#### 3.3.3 Organics/inorganics hybrid sonosensitizers

Despite the excellent acoustic dynamic effect of organic molecular sonosensitizers, the majority of these macromolecules are hydrophobic and prone to aggregation in solution, resulting in a self-quenching phenomenon. Furthermore, inorganic sonosensitizers may present biosafety concerns due to their inability to be degraded *in vivo* (134). In order to address the limitations of both organic and inorganic sonosensitizers, hybrid sonosensitizers, which combine the properties of both, have been developed. Organic/inorganic hybrid sonosensitizers encompass a range of materials, including those with a mesoporous structure, yolk shell, metal porphyrin, and metal-organic framework (MOF) composition (135). Among these, MOF have emerged as a particularly promising area of research. Among the aforementioned materials, those belonging to the category of MOF nanomaterials are particularly noteworthy. From one perspective, organic ligands in MOFs have the capacity to absorb ultrasound, thereby activating metal ions/clusters through a charge transfer mechanism that links the body to the metal. This mechanism facilitates the separation of electrons and holes and enhances the generation of ROS in comparison to organic or inorganic sonosensitizers. Conversely, the porous structure of MOFs provides a periodic array that minimises self-quenching to a minimum volume and allows for rapid ROS diffusion, thereby increasing ROS generation efficiency. Furthermore, MOFs possess the attributes of a large surface area, high porosity, and facile modification, which enable the loading of diverse anticancer drugs and the integration of SDT with other tumour therapies, thus enhancing its efficacy (136–139).

To address the limitations of the organic, acoustically sensitive porphyrin, Lu and his colleagues devised a strategy wherein an Mn-porphyrin-based MOF was loaded with the immune adjuvant R848 within the porous structure of the MOF (125). This was followed by the coating of the surface with an AuPt shell through reduction and the subsequent coating of the surface with modified Hep1–6 tumour cell vesicles. In response to ultrasonic stimulation, MOF acoustic sensitizers released a substantial quantity of ROS, which induce ICD in a collaborative manner with the immune adjuvant R848, thereby triggering a highly efficacious immune response. Concurrently, MOF-mediated SDT facilitated the catalysis of AuPt shell-mediated catalytic convertor-based therapy. In addition to enhancing the targeting of tumour cells with a similar genetic make-up, the outermost tumour cell membrane also enhanced the cross-presentation of tumour antigen MHC-I/II by DCs. *In vivo* experiments demonstrate that the MOF material is capable of inducing a systemic immune response and long-term memory immunity, and of eradicating both primary and distal tumours, thereby fulfilling the function of a vaccine.

The product of the SDT is ROS. In cells, mitochondria play a pivotal role in ROS production and apoptosis control. The targeted induction of apoptosis may be achieved with greater efficacy by inducing SDT in mitochondria. Luo’s team employed the mitochondrial targeting properties of triphenylphosphine, which they coupled with a meso-tetra(4-carboxyphenyl)porphine (H_2_TCPP) acoustic sensitised agent, utilising it as an organic ligand of MOF (126). A metal complex of Zr was formed with the organic ligand, resulting in the synthesis of an MOF that was loaded with the immune adjuvant imiquimod (R837). In order to achieve homologous targeting, the final material was formulated with a 4T1 cell membrane. The highly effective SDT of MOFs induces the *in situ* release of tumour-associated antigens, which results in a stronger vaccine-like activity and a robust immune response when combined with immunoadjuvants.

## 4 Conclusion

The use of ultrasound in clinical care has increased significantly in recent years due to the non-invasive and highly penetrating nature of this technology (140). In the context of tumour therapy, ultrasound has the potential to serve not only as a direct ablation method for tumour tissue, but also as an immune system activator (141–143). This is achieved by the destruction of tumour-associated antigens released following tumour destruction. Moreover, through the use of cavitation ultrasound, the blood-brain barrier or vascular tumour barrier can be transiently opened, facilitating precise and effective drug delivery and thus enhancing the efficacy of tumour immunotherapy (144, 145). Another area of intense research in the field of ultrasound-assisted tumour immunotherapy is SDT, which involves the induction of immunogenic death and the initiation of an immune response through the mass release of ROS from sensitizers upon sonication. Ultrasound-assisted immunotherapy shows great promise in treating malignant tumour. However, the application of ultrasound in immunotherapy is still confronted with significant challenges. Firstly, in the SDT-induced ROS product, molecular oxygen in the tissues an important catalyst. Tumour cells grow in a hypoxia microenvironment (146), indicating that hypoxia condition in tumour microenvironment is one of the challenges limiting the production efficiency of ROS upon SDT. Therefore, alleviating hypoxia in the tumour tissues are a promising strategy in enhancing the efficacy of SDT. Jiang et al. demonstrated that on-demand oxygen delivery to the tumour site could enhance SDT-related tumour immunotherapy by inhibiting both primary and distal tumour growth (147). Additionally, the SDT-induced ROS as a stress can result in the accumulation of reducing substances such as glutathione (GSH) in the tumour tissues to possibly weaken the SDT efficacy. Tian and their colleagues found that GSH inhibitors significantly increased the immunogenic death of tumour cells by SDT (148). These results have demonstrated that the further improvements of TEM, especially hypoxia and redox state, have huge potentials in improving the SDT-assisted immunotherapy on tumours. Secondly, the development of a safe and highly efficient sonosensitizers is still a hotspot to achieve SDT-related tumour immunotherapy. Although the different types of sonosensitizers including organic sonosensitizer, inorganic sonosensitizer and organics/inorganics hybrid sonosensitizers have been widely studied and developed, currently the majority of sonosensitizers are developed based on photosensitizers as prototypes, and there are few established guidelines for the development of efficacious, safe and reliable sonosensitizers (149). Their hydrophobicity and poor targeting are also challenges. Along with the rapid advance of intelligent and responsive nanoscale in addressing the targeting, efficacy and hydrophobicity of drugs, the development of targeted, smart and responsive nanosonosensitizers will be a promising future direction in the field of SDT-assisted immunotherapy (150).

Moreover, the optimal setting of ultrasound parameters should be reckoned with in performing ultrasound-assisted immunotherapy on tumours. The parameters of ultrasound determine the effect of thermal or mechanical action. In practical applications, both effects often occur simultaneously. Therefore, further research is required to determine the optimal parameters for immune activation, with a particular focus on the underlying mechanisms of ultrasound. Furthermore, although the long-term clinical application of ultrasound has demonstrated its excellent safety profile, the current basic and clinical research on ultrasonic parameters lack a unified standard. It is therefore essential to conduct further research into the intrinsic characteristics of ultrasound parameters and identify the factors that influence them. This will enable the gradual establishment of a scientific, reasonable and widely accepted standard system, providing a more robust theoretical basis and scientific evidence for the clinical application of ultrasound technology. As a result of the increasing depth and breadth of research into SDT, it is anticipated that these studies will facilitate the imminent transition of ultrasound-assisted tumour immunotherapy into clinical practice.
